# Is Child Maltreatment Painful? An Exploration of Peritraumatic Pain in Child Maltreatment

**DOI:** 10.1007/s40653-024-00682-x

**Published:** 2025-01-04

**Authors:** Noga Tsur, Carmit Katz, Nofar Shemesh

**Affiliations:** 1 Bob Shapell School of Social Work, Tel Aviv University, Tel Aviv, Israel; 2 The Paul Baerwald School of Social Work and Social Welfare, The Hebrew University of Jerusalem, Jerusalem, Israel; 3 The Haruv Institute, The Haruv Institute, Jerusalem, Israel

**Keywords:** Child maltreatment, Child abuse, Sexual abuse, Physical abuse, Emotional abuse, Pain, Peritraumatic

## Abstract

Substantial findings point to the risk of chronic pain following child maltreatment (CM). However, a coherent explanation for the mechanisms underlying pain following CM is lacking. Although peritraumatic pain may encompass a significant role in these processes, a systematic quantification of peritraumatic pain in CM has never been conducted. This study was conducted to provide an initial exploration of peritraumatic pain characteristics in CM, and its links with CM characteristics, pain expression, and later posttraumatic outcomes. Two samples of adult CM survivors (*N* = 160 and *N* = 120, respectively) filled out self-report questionnaires assessing peritraumatic pain characteristics, CM characteristics, peritraumatic dissociation, and complex posttraumatic-stress symptoms (cPTSD). Peritraumatic pain was reported by 42.2% (76 participants) and 57% (69 participants) in Samples 1 and 2, respectively. While pain was most prevalent in physical abuse (90%), it was also prevalent in sexual (63%) and emotional abuse (37.5%; *X*^*2*^(3) = 14.65, *p* = .002). Peritraumatic pain was most prevalent when the perpetrator was a family member (*X*^*2*^(3) = 14.65, *p* = .002), longer CM duration (*p* < .001), and in fight-or-flight peritraumatic responses (*X*^*2*^(2) = 7.13, *p* = .028). Peritraumatic pain expression did not differ for CM type (*p* > .083), and most participants reported that the perpetrator ignored their pain (73.9%). Explicit and concealing expressions of pain were associated with later cPTSD symptoms (*p* < .047). The findings provide an initial understanding of peritraumatic pain characteristics and phenomenology, demonstrating that pain is a substantial constituent of CM experiences and potential posttraumatic outcomes.

## Introduction

Extensive research has documented the malevolent long-lasting implications of child maltreatment (CM) for survivors, pointing to multiple physical and psychosocial health-related outcomes (Hailes et al., 2019; Irish et al., 2010). One particular implication of CM has been seen in regard to reports of unexplained pain, chronic pain, as well as multiple pain-related morbidities, which have shown to be highly prevalent among CM survivors (Nicol et al., 2016; Peles et al., 2016; Sachs-Ericsson et al., 2007; Spiegel et al., 2016). This link between exposure to interpersonal trauma and chronic pain has been extensively assessed, yielding findings demonstrating a significant association between CM and headaches (Lee Peterlin et al., 2007), irritable bowel syndrome (Beesley et al., 2010; Ross, 2005), fibromyalgia (Häuser et al., 2014), interstitial cystitis/painful bladder syndrome (Mayson & Teichman, 2009), genito-pelvic pain (Santerre-Baillargeon et al., 2017), and other chronic pain conditions (Walsh et al., 2007). Notably, a systematic literature review assessing longitudinal indications of the CM-chronic pain link revealed the low quality of prospective examinations (Marin et al., 2021).

Substantial empirical efforts have been dedicated to uncovering the underlying mechanisms of the close link between trauma exposure, posttraumatic stress disorder (PTSD), and chronic pain. Such investigations have highlighted the role of PTSD as a precursor to chronic pain following CM (Marin et al., 2021). Previous investigations have pointed to mutual maintaining factors (Sharp & Harvey, 2001) and shared vulnerabilities (Asmundson et al., 2009), which exacerbate both PTSD and chronic pain. Such mutual maintaining factors refer to physiological, affective and behavioral components of PTSD and pain, which mutually maintain or intensify each other (Asmundson & Katz, 2009; Sharp & Harvey, 2001). The shared vulnerabilities point to the interaction of psychological vulnerability to loss of control, lower physiological threshold, and the exposure to trauma as factors yielding a higher vulnerability to PTSD and pain and their mutual maintaining factors (Asmundson & Katz, 2009). Recent explanations have uncovered a unique paradoxical pain profile in PTSD (Defrin et al., 2015), as well as inflammation related to impediments of immune system functions (Mayson & Teichman, 2009; Nicolson et al., 2010), pointing to physiological underlying mechanisms tying together pain and PTSD**.**

Nevertheless, a recent literature review revealed that one fundamental framework related to the CM-pain link has rarely been assessed to date (Tsur et al., 2023). This framework refers to the exploration of pain sensations as experienced within CM victimizing acts, i.e., peritraumatic pain. Thus, it appears that the question of whether CM is a painful experience has scarcely been systematically assessed (Tsur et al., 2023). Only a few studies have provided preliminary insights into this phenomenon. For example, two studies have demonstrated the phenomenology of peritraumatic pain in **child** sexual abuse (Tsur et al., 2020b) and child physical abuse (Tsur et al., 2020a). These examinations revealed that children reported high intensity pain sensations during victimizing acts. However, it appears that these pain sensations are counterbalanced by the children themselves, who relate to it as “It was so painful… not so much, just a little” (Tsur et al., 2020a, p. 4). Peritraumatic pain was also found to be counterbalanced by the perpetrator: “He [the perpetrator] said, ‘‘cause your body is developing, that is why it hurts.’” (Tsur et al., 2020b, p. NP44***1***0***1***3), reflecting essential subjectivity demolishment, inherently woven within the child-perpetrator bond (Herman, 2015). Although these findings underline peritraumatic pain as a significant manifestation within the complex and multifaceted fabric of CM, a quantitative systematic assessment is still lacking. As such, the relevance of peritraumatic pain for the trauma-pain link is unknown.

Another relevant question is what relevance does peritraumatic pain in CM hold for later implications and outcomes? Unsurprisingly, considering the paucity of research on peritraumatic pain, very little is known about this matter. Still, some findings may pave the way for initial insights. Findings stemming from studies on adult torture survivors have shown that those who experienced falanga (beating of the soles of feet) were at a higher risk of developing neuropathic pain in their feet (Olsen et al., 2006; Thomsen et al., 2000), while those who were suffocated developed chronic headaches (Olsen et al., 2007). Potential explanations for these findings have been seen in other findings, demonstrating that peritraumatic pain reported by adults who experienced child abuse may be re-experienced through pain flashbacks, potentially increasing the risk of chronic pain (Tsur, 2023). Furthermore, a recent study with young adult women demonstrated that intrusive pain sensations may be conditionally induced (Franke et al., 2022), establishing support for the role of peritraumatic pain for later posttraumatic symptoms and chronic pain. Similarly, other explanations point to autobiographical pain memories as playing a potentially significant role in the link between acute post-surgical pain and later chronic pain in children (Wasiman et al., 2022). While referring to medical procedures, such mechanisms might also be relevant for understanding chronic pain following CM.

Other findings further demonstrate that peritraumatic pain might be linked to later PTSD, which, considering their comorbidity, might also take part in the development of chronic pain. For example, in young children who experienced burns, peritraumatic pain was linked with the level of acute stress responses, mediated by the parent’s stress symptoms (Stoddard et al., 2006). Other findings among children who experienced burns (Saxe et al., 2005) and adults who experienced a traumatic injury (Norman et al., 2008) and orofacial injury (Glynn et al., 2007) showed that peritraumatic pain was associated with increased risk of later PTSD. Although these findings do not refer to CM, it should be considered that some of the injuries included in the aforementioned studies may involve some sorts of interpersonal trauma and/or CM.

The current study was conducted to fill this research gap. The aim of this study was set to provide an initial exploration of peritraumatic pain in CM. Main research questions focused on ‘What is the prevenance of peritraumatic pain reports among CM survivors?’ It was hypothesized that the frequency of peritraumatic pain reports will differ among types of CM, with physical abuse demonstrating the highest prevalence of peritraumatic pain reports. The second research question focused on ‘How is peritraumatic pain manifested within the perpetrator-child dynamic, and how are these manifestations associated with later mental health? Considering the absence of previous investigations, no specific hypotheses are presented. Two samples of adult CM survivors participated in this study, responding to a survey that quantified peritraumatic pain characteristics. The survey for Sample 1 focused on CM characteristics and how they were related to the report of peritraumatic pain. The survey for Sample 2 focused on the associations between peritraumatic pain characteristics and interpersonal dynamics with the perpetrator, as well as posttraumatic symptomatology.

## Methods

This study included two samples, both of which were part of larger studies examining the implications of trauma on bodily perceptions. Sample 1 included 160 women who reported being sexually abused in their childhood. To be included in the study, participants had to (a) be between the ages of 18 to 60, (b) be fluent in Hebrew, and (c) report experiencing child sexual abuse. Data were collected via social media platforms, such as Facebook and WhatsApp, using a snowball technique. The advertising of this study explicitly invited adults who experiened childhood sexual abuse. Sample 2 included a sample of 120 adult participants (105 female and 12 male) from a larger community sample, who reported experiencing sexual abuse, physical abuse, and/or emotional abuse in their childhood. The reports of CM were based on participants’ answers to an open question from the International Trauma Questionnaire (ITQ; Cloitre et al., 2018, see details below), where participants are asked to describe the most significant traumatic experience they endured. Participants who described any form of CM were included in the current study. To be included in this study, participants had to (a) be between the age of 18 to 60, (b) be fluent in Hebrew, and (c) report experiencing child sexual, physical, and/or emotional abuse. Like Sample 1, data were collected via social media platforms, such as Facebook and WhatsApp, using a snowball technique. Participants who wished to participate, were referred to an online questionnaire via Qualtrics. Online informed consent was collected from all participants preceding access to questionnaire. The study was approved by the Institutional Review Board.

## Measures

### Sample 1 Measures

The survey of Sample 1 included questionnaires and specific questions related to CM type and intensity, peritraumatic responses, and peritraumatic pain (68 items altogether).

*Child maltreatment* was measured using the Childhood Trauma Questionnaire (CTQ; Bernstein et al., 2003). The CTQ consists of 28 items that refer to five maltreatment forms, namely, sexual abuse, physical abuse, emotional abuse, physical neglect and emotional neglect. This study utilized the physical abuse, sexual abuse, and emotional abuse (and not neglect) subscales. Participants were asked to rate whether the items were true for them on a 5-point Likert-type scale, with responses ranging between 1 (never true) to 5 (very often true). Examples of the items include “hit severely enough that bruises were noticed” (physical abuse), “was touched sexually” (sexual abuse), and “was called names by family members” (emotional abuse). Three different scores, reflecting the three forms of abuse were calculated. Previous findings have determined the scales’ validity (Bernstein et al., 2003), as well as the Hebrew version (Talmon & Ginzburg, 2017). Cronbach’s α s for the current sample were good; 0.83 for physical abuse, 0.83 for sexual abuse, and 0.89 for emotional abuse.

Additionally, information regarding specific CM characteristics were included. Participants who experienced more than one CM incidence were instructed to refer to the CM experience that was most significant to them. Information regarding the perpetrator’s identity was collected via an open question (“Who was the person from which you experienced CM?”). Participants were also asked to report their age when CM initiated (“How old were you when CM started?”), and CM duration (“For how long did you experience this CM? ).

*Peritraumatic responses to CM* were measured using the Multifaceted Peritraumatic Responses to Child Abuse inventory (MPR-CA; Katz et al., 2021). The MPR-CA was designed to assess four categories of peritraumatic responses to CM: automatic, behavioral, affective, and cognitive. The automatic and behavioral responses were included in this study, as they hold significance for later psychopathology (Tsur et al., 2021). The automatic category of peritraumatic responses includes three different classes of responses: (a) a tendency to freeze and/or dissociate, reflecting high levels of freeze responses and dissociation; (b) a tendency of fight and/or flight, endorsing high levels of fight and flight responses alongside high levels of physiological responses; and (c) an absence of automatic responses, reflecting low levels of all automatic responses.

The behavioral category of peritraumatic responses assesses behaviors that characterize children’s responses to maltreatment, such as “I tried to call for help,” “I did what I was told,” and “I tried to make excuses in order to avoid what was happening.” The behavioral category includes three main classes of responses: (a) high survival attempts, including a high frequency of various behavioral patterns; (b) adaptation to abuse showing high adaptation, submission, obedience and adjusting behaviors; and (c) absence of behavioral responses, reflected in low levels of most behaviors. Participants were asked to report whether they enacted the peritraumatic response (yes/no). A latent class profile analysis yielded groups of classes for each category.

To quantify ***the prevalence of peritraumatic pain reports in CM***, participants were asked to report whether they experienced pain during the CM (yes/no). If the participants experienced more than one CM incident or repeated CM, they were instructed to refer to their most significant abusive experience.

### Sample 2 Measures

The survey of Sample 2 included questionnaires assessing complex posttraumatic stress symptoms, peritraumatic dissociation, peritraumatic pain, and peritraumatic pain ambiguity and pain expression (29 items altogether).

*Posttraumatic stress symptoms and disturbance in self-organization symptoms* were assessed using the International Trauma Questionnaire (ITQ; Cloitre et al., 2018). The ITQ is a self-report diagnostic measure of posttraumatic stress (PTS) and disturbances in self-organization (DSO) symptoms, as defined by the International Classification of Diseases, 11th version (ICD-11). The scale consists of six items that represent the three PTSD clusters (i.e., re-experiencing, avoidance, and sense of threat) and another six items that represent the three DSO clusters (i.e., affective dysregulation, negative self-concept, and disturbances in relationships). Participants were asked to rate the extent to which a symptom has bothered them over the past month on a Likert scale, ranging from 0 (not at all) to 4 (extremely). Examples of PTS symptoms include: “Having upsetting dreams that replay part of the experience or are clearly related to the experience;” “Avoiding internal reminders of the experience (e.g., thoughts, feelings or physical sensations);” “Being ‘super-alert,’ watchful or on guard.” Examples of complex PTSD (cPTSD) symptoms include: “When I am upset, it takes me a long time to calm down;” “I feel worthless;” and “I feel distant or cut off from people.” Previous findings revealed the scale’s validity (Cloitre et al., 2018), and the Hebrew version supported the validity of the ITQ (Ben‐Ezra et al., 2018; Gilbar et al., 2018). Cronbach’s *α* s for PTS symptoms was 0.87, and for DSO symptoms was 0.89, indicating high reliability.

Peritraumatic dissociation was assessed using the he Peritraumatic Dissociative Experiences Questionnaire (PDEQ; Marmar et al., 2004). This questionnaire is consisted of 10 items, dissociative experiences during traumatic events. Participants are asked to rate, on a 5-point Likert scale, the extent to which the item presented is true to them (1 = very true, 5 = not at all true). Example items include “Felt disorientated. . uncertain where I was,” and “Body seemed distorted or changed.” The sum of all items is calculated into a single peritraumatic dissociation score, with a higher score reflecting *lower* dissociation. Previous findings demonstrate the validity of the Hebrew version (Shalev et al., 1998). Cronbach’s *α* s for the current study was 0.92, indicating high reliability.

To quantify ***the prevalence of peritraumatic pain reports in CM and its intensity***, participants were asked to report whether they experienced pain during the CM (yes/no). If the participants experienced more than one CM incident or repeated CM, they were instructed to refer to their most significant abusive experience. *Peritraumatic pain intensity* was assessed using a Visual Analogue Scale (VAS), with 0 = no pain at all, and 10 = reflecting the worst pain one can imagine (Langley & Sheppeard, 1985).

*Peritraumatic pain ambiguity*,* and expression of peritraumatic pain* were assessed using items developed based on previous qualitative findings, as reported by children exposed to physical abuse (Tsur et al., 2020a), and sexual abuse (Tsur et al., 2020b). The pain ambiguity items reflected uncertainty of peritraumatic pain sensations. The three items included: “I experienced pain, but couldn’t say where in my body I felt it,” I experienced pain, but the sensation was vague and/or unclear,” and “I felt as if the pain was and wasn’t there.” Participants who reported experiencing peritraumatic pain were asked to rate on a 5-point Likert scale the extent to which the items were true to them (1 = not at all, 5 = very true). The average of the three items was calculate, with a higher score reflecting a higher peritraumatic pain ambiguity. Cronbach’s *α* s for the current study was 0.81, indicating good reliability.

*Peritraumatic pain expression* was assessed using two items. *Explicit expression of peritraumatic pain* was assessed with the following item: “I experienced pain, and I yelled/cried out of pain, or expressed it in another way.” *The tendency to conceal peritraumatic pain* was assessed with the following item: “I experienced pain, but didn’t show it.” Participants who reported experiencing peritraumatic pain were asked to rate on a 5-point Likert scale the extent to which the items were true to them (1 = not at all, 5 = very true), with a higher score reflecting a higher tendency to explicitly express pain, and/or to conceal peritraumatic pain.

### Data Analytical Plan

All data analyses were conducted using SPSS software (version 28). Descriptive statistics demonstrated the sample characteristics and report of peritraumatic pain prevalence. Independent sample t-test were used to explore group differences in demographic, CM characteristics, peritraumatic dissociation, and posttraumatic symptoms between participants reporting peritraumatic pain and those who reported no peritraumatic pain. Chi-square test was used to assess whether peritraumatic pain differed according to the perpetrator’s identity. Finally, two Hierarchical linear regression models were conducted to test whether peritraumatic pain intensity, explicit expression of pain, tendency to conceal pain, and peritraumatic dissociation were implicated in posttraumatic symptoms.

## Results

### Sample 1: Sample Characteristics, Peritraumatic Pain Prevalence and Abuse Characteristics

Sample 1 consisted of 160 women (mean age = 31.4; *SD* = 8.31) who reported being sexually abused during childhood. The majority of the participants were also exposed to other types of CM: 43.75% (*n* = 70) reported experiencing child physical abuse, and 25.63% (*n* = 41) reported experiencing child emotional abuse. Among the participants in Sample 1, 42.2% (*n* = 76) reported experiencing peritraumatic pain.

As presented in Table 1, an independent sample t-test revealed that reports of peritraumatic pain did not differ regarding the age of participants today, nor their age when the CM was initiated. However, it differed according to the duration of CM, demonstrating that peritraumatic pain was more prevalent in longer durations of CM. Additionally, participants who reported experiencing peritraumatic pain were also exposed to a higher intensity of sexual, physical, and emotional abuse.

**Table 1:** Sample 1 background and CM characteristics

	Peritraumatic pain *n* = 76		No Peritraumatic pain *n* = 84		t(175)	*p*
	M	SD	M	SD		
Age today	30.83	9.02	31.91	7.82	− 0.8	0.43
Age when CM initiated	7.75	4.72	8.78	3.74	−1.59	0.11
CM durance (years)	8.88	8.55	3.53	3.44	4.22	< 0.001
CTQ sexual	16.29	6.34	14.82	5.22	1.7	0.091
CTQ physical	12.8	5.71	7.24	3.11	8.29	< 0.001
CTQ emotional	17.13	5.96	11.25	5.29	6.8	< 0.001

A chi-square test was conducted to assess whether peritraumatic pain differed according to the perpetrator’s identity. As shown in Table 2, more than half of the participants who reported that the perpetrator was a family member also reported experiencing peritraumatic pain. However, peritraumatic pain was much less prevalent when the perpetrator was someone familiar to the child (not a family member) or a stranger.

**Table 2:** Sample 1 peritraumatic pain and perpetrator’s identity

	Peritraumatic pain		No Peritraumatic pain	
	n	Percent in perpetrator identity	n	Percent in perpetrator identity
Family member	51	54.3%	43	45.7%
Someone familiar	17	28.3%	43	71.7%
Stranger	4	22.2%	14	77.8%
Perpetrator identity				

X^2^(3) = 14.65, *p* = .002

## Sample 1: Peritraumatic Pain and Peritraumatic Responses to CM

Chi-square tests were conducted to assess whether peritraumatic pain differed according to peritraumatic responses to CM. As presented in Table 3, peritraumatic pain was most prevalent when peritraumatic responses were characterized by fight-or-flight responses, presumably indicating that attempts to resist the abusive acts increased the risk of peritraumatic pain. This pattern was also seen in the behavioral pattern of peritraumatic responses, with peritraumatic pain being most prevalent among participants who reported extensive behavioral acts of attempting to survive the abuse, such as adaptation, obedience, and attempts to avoid abuse.

**Table 3:** Automatic and behavioral peritraumatic responses and peritraumatic pain

	Peritraumatic pain			No Peritraumatic pain	
	n		Percent in peritraumatic response	n	Percent in peritraumatic response
***Peritraumatic automatic response***					
Tend to freeze and dissociate		26	41.9%	36	58.1%
Tend to fight and flight		26	59.1%	18	40.9%
Absence of automatic response		24	33.8%	47	66.2%
X^2^(2) = 7.13, *p* = .028					
***Peritraumatic behavioral response***					
Highly survival attempts		11	68.8%	5	31.3%
Adaptation to abuse		40	50%	40	50%
Absence of behavioral responses		25	30.9%	56	69.1%
X^2^(3) = 10.8, *p* = .005					

## Sample 2: Sample Characteristics,Peritraumatic Pain Prevalence and Abuse Characteristics

Sample 2 comprised mostly women (87.5%; *n* = 105) and 12 male participants. A chi-square test showed that male and female participants did not differ in the prevalence of peritraumatic pain (*X*^*2*^(2) = 3.23, *p* = .2). The mean age was 34 (*SD* = 10.66). Independent sample t-test revealed no significant differences in age between participants who reported experiencing peritraumatic pain and no peritraumatic pain (*t*(109) = 0.75, *p* = .45).

Among the participants in Sample 2, 57% (*n* = 69) reported experiencing peritraumatic pain. The mean peritraumatic pain intensity was 6.67 (*SD* = 3.24, range: 0–10), and the median was 8; 27.6% of the sample (*n* = 24) reported pain intensity of 10, which is the highest pain intensity score. A chi-squared test was conducted to indicate whether the type of maltreatment was implicated in a higher prevalence of peritraumatic pain. As seen in Table 4, peritraumatic pain was most prevalent in physical abuse, followed by sexual abuse. However, an ANOVA test comparing pain intensity in maltreatment types yielded an insignificant result (*F*(3,83) = 0.38; *p* = .77), demonstrating that pain intensity did not differ between CM types.

**Table 4:** Sample 2 peritraumatic pain and CM type

	Peritraumatic pain		No Peritraumatic pain	
	n	Percent in CM type	n	Percent in CM type
Sexual abuse	34	63%	20	37%
Physical abuse	18	90%	2	10%
Emotional abuse	12	37.5%	20	62.5%
Did not disclose type of CM	5	83.3%	1	16.7%

X^2^(3) = 14.65, *p* = .002

An independent sample t-test was conducted to explore group differences in peritraumatic dissociation in participants reporting peritraumatic pain and those who reported no peritraumatic pain. The results demonstrated that participants who reported peritraumatic pain also reported more peritraumatic dissociation (mean = 2.88, *SD* = 1.12; note: a lower score reflects more dissociation) compared to participants who did not report experiencing peritraumatic pain (mean = 3.58, *SD* = 1.17; *t*(76.96) = −3.02, *p* = .002).

### Sample 2: Explicit Expression of Pain, Tendency to Conceal Pain, and Pain Ambiguity

Pearson correlations were conducted to assess whether peritraumatic pain intensity was correlated with pain expression and pain ambiguity. As shown in Table 5, peritraumatic pain intensity was correlated with more explicit expressions of pain during CM incidents. However, pain intensity was not correlated with the tendency to conceal pain or pain ambiguity. As expected, reports of explicit expression of pain were inversely correlated with the tendency to conceal pain during CM. Finally, pain ambiguity was significantly correlated with the tendency to conceal pain, however, no such correlation was found with the explicit expression of pain.

**Table 5:** Sample 3 Pearson correlation between peritraumatic pain intensity, expression, and ambiguity

	*n*	M	SD	1	2	3	4
1. Peritraumatic pain intensity	87	6.67	3.24	-			
2. Explicit expression of pain	78	2.99	1.77	0.42^***^	-		
3. Concealed pain expression	78	2.91	1.68	0.15	− 0.39^***^	-	
4. Ambiguity of peritraumatic pain	80	2.31	1.25	0.14	− 0.13	0.38^***^	-

*p* < .001^***^

One-way ANOVA was conducted to compare CM types regarding peritraumatic pain ambiguity, which yielded insignificant results (*F*(2,72) = 3.1; *p* = .15), demonstrating that maltreatment type did not differ in relation to pain ambiguity during CM incidences. Two one-way ANOVAs were conducted to assess whether the expression of peritraumatic pain during abusive incidences differed between maltreatment types. Participants who did not disclose the type of maltreatment were excluded from these analyses (*n* = 6). The ANOVAs indicated that maltreatment type did not differ in relation to the explicit expression of pain (*F*(2,71) = 2.58; *p* = .083), nor the concealing of pain during maltreatment (*F*(2,71) = 2.75; *p* = .38).

Among the 69 participants who reported experiencing peritraumatic pain during maltreatment, 51 (73.9%) reported that the perpetrator ignored their pain during the CM incidences. Concurrently, only 9 participants (13%) reported that the perpetrator related to their pain during the maltreatment. Examples of perpetrators’ references to pain included: “always apologized,” “told me: you see what you make me do to you?” “told me that I deserve it,” “told me that I was not a virgin,” and “he [either] laughed or yelled in anger.”

### Sample 2: Peritraumatic Pain and Posttraumatic Stress Symptoms

An independent sample t-test was conducted to **explore group differences in PTS and DSO symptoms in participants reporting peritraumatic pain and those reporting no peritraumatic pain.** The results demonstrated that participants who reported peritraumatic pain reported more PTS symptoms (mean = 3.11, *SD* = 1.15) compared to participants who did not report peritraumatic pain (mean = 2.48, *SD* = 0.97; *t*(110) = 3.02, *p* = .003). Participants who reported peritraumatic pain also reported more DSO symptoms (mean = 3.43, *SD* = 1.05) compared to participants who did not report experiencing peritraumatic pain (mean = 2.91, *SD* = 1.19; *t*(110) = 2.46, *p* = .016).

Next, two hierarchical linear regression models were conducted to assess the role of peritraumatic pain characteristics for PTS symptoms, with PTS and DSO symptoms as outcome variables. Peritraumatic pain intensity, explicit expression of pain, tendency to conceal pain, and peritraumatic dissociation were the independent variables. The regression model predicting PTS symptoms was significant, explaining 32% of the shared variance of PTS symptoms. As presented in Table 6, pain intensity was not associated with later PTS symptoms. However, explicit expression of pain and the tendency to conceal pain were significantly correlated with PTS symptoms, demonstrating that the higher tendency of both pain expressions was correlated with PTS symptoms. Additionally, peritraumatic dissociation was also significantly correlated with PTS symptoms.

**Table 6:** Sample 2 regression models for predicting PTS and DSO symptoms

	PTS symptoms				DSO symptoms			
	B	SE B	ß	*p*	B	SE B	ß	*p*
Peritraumatic pain intensity	− 0.017	0.047	− 0.041	0.73	0.004	0.05	0.01	0.94
Explicit expression of pain	0.17	0.082	0.25	0.047	0.12	0.086	0.2	0.17
Concealed pain expression	0.18	0.081	0.27	0.029	0.18	0.085	0.29	0.04
Ambiguity of peritraumatic pain	0.098	0.11	0.11	0.38	0.026	0.12	0.032	0.82
Peritraumatic dissociation	− 0.41	0.12	− 0.42	< 0.001	− 0.17	0.12	− 0.19	0.17

Adjusted R^2^ = 0.32; *p* < .001

Adjusted R^2^ = 0.093; *p* = .04

The regression model predicting DSO symptoms was significant, explaining 9.3% of the shared variance of DSO symptoms. Pain intensity was not associated with later DSO symptoms. The only significant predictor of DSO symptoms was the tendency to conceal pain during CM. No other variables were significantly correlated with DSO symptoms.

## Discussion

This study’s findings portray an initial systematic exploration of peritraumatic pain in CM. The findings demonstrated that about half of the CM survivors reported experiencing pain during CM, which was linked to higher CM duration and severity. The findings further outlined new indications regarding the implications of the explicit expression of peritraumatic pain during CM incidents, as well as the experience of pain ambiguity. Finally, the findings provided insights into the role of peritraumatic pain expression for later posttraumatic symptoms.

This study underlines several CM characteristics in which peritraumatic pain is the most prevalent. While survivors of physical abuse reported the highest rates of peritraumatic pain, most sexual abuse survivors also reported experiencing peritraumatic pain, as well as almost 40% of emotional abuse survivors. Pain intensity, however, did not differ between maltreatment types. The findings further highlighted that peritraumatic pain was more prevalent among participants who reported peritraumatic fight-or-flight responses and those who tended to actively try to end the maltreatment (i.e., high survival peritraumatic response). Recent findings have uncovered a new framework of peritraumatic responses to CM. These studies have demonstrated that the traditional fight-flight-freeze model has not been inclusive of the various peritraumatic responses to maltreatment and that children’s responses to maltreatment vary on the automatic, behavioral, affective, and emotional levels (Katz et al., 2021; Katz, Tsur, Katz et al., 2020a, b; Tsur et al., 2021). Combined with the current findings, it is apparent that children who actively attempt to resist the maltreatment act (either by adapting and complying, trying to avoid the CM, fighting or “flighting”) are prone to experience more peritraumatic pain than children who do not respond in a similar manner. It is possible that this dynamic could be explained by the child’s age or maltreatment duration, with an older age or longer CM duration yielding more resistance and, consequently, more pain. These findings demonstrate that pain is an inherent component of maltreatment experiences and should be empirically and clinically targeted as such.

Peritraumatic dissociation has been documented in the literature (e.g., Marmar et al., 2004), demonstrating mixed findings as to its implications for posttraumatic aftermath, with some demonstrating its potential link with later distressing intrusions (Danbock et al., 2021) and others revealing inconsistent findings regarding its implications for posttraumatic symptoms (Beutler et al., 2022; Memarzia et al., 2021). Notably, the findings demonstrate that peritraumatic dissociation was shown to be association with a higher prevalence of peritraumatic pain. Previous findings have shown that dissociation is often linked with reduced pain sensitivity in participants with borderline personality disorder (Ludäscher et al., 2007, 2010), and participants with PTSD (Defrin et al., 2015). Although these findings may contradict the current findings, suggesting that participants reporting peritraumatic dissociation should have experienced less pain. Presumably, one explanation is that during victimizing acts, mental and physical states may fluctuate, encompassing some moments of dissociation, which may vary in their intensity. Additionally, considering that peritraumatic dissociation reflects a defense mechanism, aimed at promoting mental survival in an unbearable experience (Marmar et al., 2004; Spiegel, 1988), it is possible that dissociation was activated by harsh victimizing acts that involved peritraumatic pain. In such situations, peritraumatic pain may instigate peritraumatic dissociation, and therefore may be reported in sync.

Literature has long spotlighted the complex and unique dynamics between perpetrators and victims, revealing the characteristics of this perverse bond (Herman, 2015). One significant element of this dynamic is the inclusion of dependence, love, and intimate feelings, which are twistedly interlocked with the comprehensive rebuttal of the child’s subjectivity (Katz et al., 2020a; Lassri et al., 2022; Tener, 2021). The child’s endeavor to survive a relationship encompassing such contradicting poles may yield a distorted experience of self and self-with-others (D’Andrea et al., 2012; Ferenczi, 1933; Herman, 2015), often worsening throughout adulthood (Cloitre et al., 2009; D’Andrea et al., 2012; Tsur et al., 2021). Considering the evolutionary role of acute pain as a signal of danger, reflecting actual or potential tissue damage (Stevens, 2021), peritraumatic pain may be considered an internal subjective signal for the child, a warning that “something wrong is taking place.” The current findings revealed that within the twisted dynamic between the perpetrator and child, this signal has been distorted – both by the child and the perpetrator.

Indeed, the findings showed that most participants reported that the perpetrator ignored and did not respond to the pain they experienced. The perpetrators who did respond reinforced the subjectivity-diminishing dynamic. Additionally, the participants varied in the extent to which they expressed their pain during the CM incidents, revealing both a tendency to explicitly express their pain sensations as well as a tendency to conceal them. High pain intensity was associated with the explicit expression of pain. However, the tendency to conceal pain was not associated with pain intensity, suggesting that some children do not express severe experiences of pain when they are abused. Previous findings have supported this notion, revealing that children are capable of hiding facial expressions of pain, and that their parents demonstrate difficulty identifying such expressions (Larochette et al., 2006). Similar to the children instructed to fake facial expressions of “no pain” in Larochette et al.’s (2006) study, it is possible that the unique perpetrator-child dynamics indicate to children that they should avoid expressing their pain. This understanding may reflect the internalization of the abusive dynamic by the child.

Unsurprisingly, the findings also showed that the expressions of peritraumatic pain, either explicit or concealing, were associated with later psychopathology. That is, while peritraumatic pain intensity was not associated with later psychotypology, PTS symptoms were associated with both explicit and concealed pain expressions. These findings may suggest that it is not the pain sensation per se that is implicated in later PTS symptoms, but rather the ways in which it is dealt with within the relationship with the perpetrator. DSO symptoms, however, were only associated with the concealing of peritraumatic pain. These findings are in line with the extensive literature underlining the malevolent effects of abusive relationship dynamics for cPTSD (e.g., Cloitre et al., 2009; Dorahy et al., 2016; Dorahy & Clearwater, 2012; Van der Kolk, 2017). Additionally, findings stemming from research assessing the role of parent-child perceptions of pain have demonstrated the significance of parent-child reminiscing regarding the ways children remember their pain, thus reinforcing the ways pain is approached within a relationship dynamic (Noel et al., 2019). Aligning these understandings with the current findings, it appears that pain may reflect a microcosm of the perpetrator-child dynamic and, therefore, should be further explored in research and practice.

One significant and interesting finding can be seen in the report of peritraumatic pain sensations among the participants exposed to emotional abuse. Considering that emotional abuse generally does not involve physical victimization, no significant tissue damage is expected to take place. Pain researchers might suggest that, in the absence of nociceptive stimuli, it is impossible that the participants experienced actual physical pain. Indeed, the report of peritraumatic pain in emotional abuse may reflect an experience related to emotional suffering, presumably confusing it with physical pain sensations. The revised definition of pain proposed by the International Association of the Study of Pain is: “An unpleasant sensory and emotional experience associated with, or resembling that associated with, actual or potential tissue damage” (Raja et al., 2020). Therefore, it is possible that stress related sensations, paired with emotional suffering, could have produced pain-like sensations. Otherwise, considering the potential bias in retrospective reports, it may also be that memory distortions affected the report of peritraumatic pain. Another possibility could be that during the victimizing act, children may react physically in a way that causes nociceptive pain. For example, while stressed, a child may grab their leg or hand, push themselves powerfully into nearby furniture, or experience high muscle tension that might evoke pain. Taken together, this perplexing finding calls for further research to uncover empirically supported explanations.

The current findings provide new insights for clinical efforts with CM survivors. First, and simply put, the findings indicate that experiences of pain should be targeted in clinical settings, as essential component of the maltreatment experience. Considering that pain may be re-experienced in intrusive pain symptoms (Franke et al., 2022; Tsur, 2023), which may be linked with pain chronicity, it is recommended that new interventions will be developed that may alleviate these processes, and potentially reduce the risk of posttraumatic chronic pain. Finally, clinicians are encouraged to embrace the understanding of pain as a biopsychosocial phenomenon (Hadjistavropoulos et al., 2011; Noel et al., 2009), thus relevant not only in medical settings, but also in psychosocial interventions.

The limitations of this study should be acknowledged when considering its findings. First, although all of the participants are adult CM survivors, the findings are based on retrospective reports of their experiences that took place many years earlier. Therefore, the report of peritraumatic pain may be affected by memory processes. Furthermore, the findings may be biased by the convenience sampling procedure and small sample sizes, proposing barriers to the ability to generalize the findings among adults who endorsed CM in general. Considering that peritraumatic pain has scarcely been examined previously, the current study utilized measures with limited validity. Future research should invest in developing valid and reliable assessment tools to quantify peritraumatic pain, in general, and among children, in particular. Finally, one particular issue that should be considered is that the report of peritraumatic pain may be related to the CM severity. Future studies should quantify the type and severity of maltreatment acts that are more prone to be implicated in peritraumatic pain.

Is CM painful? This study provides a clear and straightforward answer to this question, showing that, in about half of the cases, the answer is “yes.” While peritraumatic pain is more prevalent among some CM types and characteristics, pain seems to be woven into the abusive CM dynamic and experience as a whole. The findings of this study provide a new roadmap for the study of peritraumatic pain in CM, emphasizing its applicability as a core ingredient of the CM experience.
